# Hydrogen placement in protein-ligand complexes under consideration of tautomerism

**DOI:** 10.1186/1758-2946-3-S1-P13

**Published:** 2011-04-19

**Authors:** S Bietz, S Urbaczek, M Rarey

**Affiliations:** 1 ZBH – Center for Bioinformatics, University of Hamburg, Bundesstr. 43, 20146 Hamburg, Germany

## 

Protein-ligand complexes are often consulted for the understanding of binding modes and mechanisms of action as well as the development of novel drugs. Unfortunately the resolution of most x-ray structures is too low to resolve hydrogen atoms. However, hydrogen positions play a major role in the analysis of important interaction types as hydrogen bonding or metal interactions. Therefore, it is important to predict the orientation of hydrogen containing rotatable groups as well as sensible protonation and tautomeric states of both protein and ligand. While in most cases these degrees of freedom are still manageable for the protein and therefore incorporated in the models of the most common prediction tools for hydrogen placement [1-3], the consideration of different protonation states and tautomers of the ligand and their relative frequency can, due to chemical multiplicity and the physicochemical complexity of protonation and tautomeric equilibria, easily become a complicated problem.

We present a new method for the prediction of hydrogen positions in protein-ligand complexes that considers tautomeric variability of the ligand in addition to common degrees of freedom. Beginning with a random tautomeric state, different reasonable tautomers of the ligand are enumerated and their relative stability is estimated on the basis of a heuristic scoring scheme. This tautomerism model is integrated in the hydrogen placement application Protoss [3].

Our approach permits an enhanced automatic prediction of hydrogen positions, especially for ligands that exhibit tautomers with similar stability but different interaction facilities. Furthermore we were able to reproduce the ligand tautomers that were proposed in several studies of tautomerism preferences in protein-ligand complexes.
